# Extramural vascular invasion nomogram before radical resection of rectal cancer based on magnetic resonance imaging

**DOI:** 10.3389/fonc.2022.1006377

**Published:** 2023-03-09

**Authors:** Lianfen Tian, Ningqin Li, Dong Xie, Qiang Li, Chuanji Zhou, Shilai Zhang, Lijuan Liu, Caiyun Huang, Lu Liu, Shaolu Lai, Zheng Wang

**Affiliations:** ^1^ Department of Radiology, Guangxi Medical University Cancer Hospital, Nanning, Guangxi, China; ^2^ Department of Radiology, The First Affiliated Hospital of Guangxi Medical University, Nanning, Guangxi, China

**Keywords:** nomogram, rectal cancer, magnetic resonance imaging, extramural vascular invasion, tumor markers

## Abstract

**Purpose:**

This study verified the value of magnetic resonance imaging (MRI) to construct a nomogram to preoperatively predict extramural vascular invasion (EMVI) in rectal cancer using MRI characteristics.

**Materials and methods:**

There were 55 rectal cancer patients with EMVI and 49 without EMVI in the internal training group. The external validation group consisted of 54 rectal cancer patients with EMVI and 55 without EMVI. High-resolution rectal T2WI, pelvic diffusion-weighted imaging (DWI) sequences, and dynamic contrast-enhanced magnetic resonance imaging (DCE-MRI) were used. We collected the following data: distance between the lower tumor margin and the anal margin, distance between the lower tumor margin and the anorectal ring, tumor proportion of intestinal wall, mrT stage, maximum tumor diameter, circumferential resection margin, superior rectal vein width, apparent diffusion coefficient (ADC), T2WI EMVI score, DWI and DCE-MRI EMVI scores, demographic information, and preoperative serum tumor marker data. Logistic regression analyses were used to identify independent risk factors of EMVI. A nomogram prediction model was constructed. Receiver operating characteristic curve analysis verified the predictive ability of the nomogram. *P* < 0.05 was considered significant.

**Result:**

Tumor proportion of intestinal wall, superior rectal vein width, T2WI EMVI score, and carbohydrate antigen 19-9 were significant independent predictors of EMVI in rectal cancer and were used to create the model. The areas under the receiver operating characteristic curve, sensitivities, and specificities of the nomogram were 0.746, 65.45%, and 83.67% for the internal training group, respectively, and 0.780, 77.1%, and 71.3% for the external validation group, respectively.

**Data conclusion:**

A nomogram including MRI characteristics can predict EMVI in rectal cancer preoperatively and provides a valuable reference to formulate individualized treatment plans and predict prognosis.

## 1 Introduction

Extramural vascular invasion (EMVI) of rectal cancer refers to the occurrence of cancer thrombi in the vascular wall or lumen outside the muscularis propria of the intestine. EMVI affects treatment planning, treatment efficacy, and prognosis (1). For patients with locally advanced rectal cancer, magnetic resonance imaging (MRI) assessment of EMVI positivity is an important indicator of poor prognosis. Compared to rectal patients without EMVI based on preoperative MRI, patients with positive mrEMVI had a fourfold risk of metachronous metastases after surgery and a decreased overall survival (2). Therefore, the EMVI status on MRI is an independent risk factor in patients with rectal cancer. This feature is a stronger predictor of distant metastasis than other morphologic features of the tumor observed by MRI. Preoperative EMVI positivity in localized advanced rectal cancer also significantly increases the risk of recurrence and metastasis after radical surgery (3, 4). The National Comprehensive Cancer Network (NCCN) and the European Society of Medical Oncology (ESMO) include baseline EMVI status as a risk stratification factor in patients with rectal cancer (5). Therefore, baseline pelvic MRI examination to evaluate EMVI status in patients with rectal cancer has become an important part of a standardized imaging evaluation report (6).

For rectal cancer patients with EMVI status, the original low-signal vascular shadow on MRI is replaced by a moderate tumor signal (i.e., a continuous extension of the tumor signal in the vascular structure outside the intestinal wall), and the tumor signal leads to continuous or discontinuous vascular expansion (7). The Mercury research group has proposed a 5-point MRI scoring system to evaluate EMVI in rectal cancer: 0: the mass penetrates the rectum wall but is smooth externally with no adjacent vessels, and MRI evaluation; 1: the mass extends in strips through the rectum wall, no adjacent vessels; 2: the mass extends in strips through the rectum wall, adjacent blood vessels, but no tumor-like signal was found in the vascular lumen, MRI evaluation; 3: the mass extends in strips through the rectum wall, adjacent blood vessels showed moderate tumor-like signal in the lumen, lumen widening, and MRI evaluation; 4: a moderate tumor-like signal in the lumen of the adjacent large vessels (above/middle/lower rectal veins) with an irregular vascular outline, MRI evaluation (8). A 5-grade scoring system using MRI to predict EMVI was created. Scores of 0–3 are defined as mrEMVI-negative (mrEMVI-), and scores of 3–4 are defined as mrEMVI-positive (mrEMVI+) (9). Many researchers have used this scoring system to preoperatively evaluate the EMVI status of rectal cancer alongside imaging features, serological tumor markers, pathological and genetic tests, and other features, but the results are inconsistent. Further, some of these methods are invasive, and we must consider the accuracy and sensitivity of diagnosis (10, 11). Therefore, identifying, convenient, and accurate methods to predict EMVI in rectal cancer is worthwhile.

A nomogram uses multi-factor regression analyses, such as logistic regression and Cox regression analyses, to integrate predictive variable indexes into a model. These indexes are given values proportional to the contribution of each variable to the event (12, 13). Each potential outcome for a given variable in the model has a corresponding score, and the total score is calculated by adding each variable’s value. Because the nomogram is a visual graph, it has a user-friendly interface and can be used to intuitively assess the results of the prediction model, helping to predict the probability of a clinical event. In clinical practice, nomograms are often used to predict the probability of specific disease-related results, such as metastasis and postoperative recurrence (14, 15). The concept of precision medicine has increased the advantages of nomograms and created greater space for their application. Nomograms for predicting anastomotic fistula, liver metastasis, and survival after rectal cancer surgery have been reported (16–20). However, there are few studies assessing nomograms for preoperative prediction of EMVI in rectal cancer. Therefore, the aim of this study was to use 3.0T MRI imaging characteristics combined with clinical and tumor markers to construct a nomogram to evaluate baseline EMVI status in rectal cancer patients. We used to MRI imaging characterize and clinical -related indicators because it has high clinical significance. Hence, this nomogram will provide valuable reference information for formulating individualized treatment plans and evaluating prognosis.

## 2 Materials and methods

### 2.1 Patient data

This retrospective study was approved by the Ethics Review Committee of Hospital institution, and the informed consent requirement was waived. Clinical and imaging data of 104 patients with rectal cancer who underwent radical resection in our hospital between January 2018 and June 2019 were collected. This cohort was the internal training group. Additionally, 109 rectal cancer patients treated at another hospital between July 2019 and December 2020 were included as an external validation cohort. This second cohort consisted of 55 men and 54 women, aged 31–83 years, with a median age of 59.7 years.

The inclusion criteria were: rectal cancer confirmed by clinical digital rectal examination and colonoscopic pathological examination without obvious bleeding obstruction, perforation, or other conditions; informed consent was previously obtained from the patient to maintain data for research; no contraindications for MRI and the successful completion of rectal MRI examination; total mesorectal excision was performed within 1 week after MRI examination and postoperative pathological data were clear and complete; and no previous history of pelvic or rectal surgery or antitumor therapy.

The exclusion criteria were: a history of other malignant tumors; preoperative radiotherapy or chemotherapy and tumor palliative surgery; incomplete clinical or imaging data or poor image quality; and contraindications to MRI, such as contrast agent allergy and renal insufficiency.

We collected information on patient age, sex, preoperative tumor markers (e.g., carcinoembryonic antigen [CEA], carbohydrate antigen 19-9 [CA19-9], cancer antigen 125 [CA125], cancer antigen 153 [CA153], and alpha fetoprotein [AFP], height, weight, and family history. All data were collected at the same time point.

### 2.2 MRI technique and imaging acquisition

The MRI field strength, scanning sequence setting, and scanning parameter setting-up of the two research institutions are similar. The MRI examination process of rectal cancer patients was carried out according to the following procedure: A DISCOVERY MR750W 3.0T scanner (GE Corporation, Boston, MA, USA) and Siemens Magnetom Trio Tim 3.0TMR scanner (Siemens AG,Munich, Germany) with a 16-channel body phase-controlled front coil were used to perform the MRIs. The scanning area comprised the entire pelvic cavity. Intestinal preparation was required before the examination, consisting of a liquid diet on the day before the examination, fasting on the day of examination, and a 10-mg 654-2 intramuscular injection 15 minutes before the examination to inhibit gastrointestinal peristalsis. The patient was placed in the supine position during the examination. Using the symphysis pubis as the coil center, the phased array coil was placed in the front and back of the patient’s pelvic cavity and kept stable and as close to the pelvis as possible. The lower abdomen and pelvic areas were fixed with a bandage to avoid artifacts caused by large breathing movements.

A DISCOVERY MR750W 3.0T scanner: Rapid spin echo was used in the routine pelvic scanning sequence. The following sequences were used: rectal high-resolution oblique T2WI (TR/TE = 6848/102 ms, layer thickness/layer spacing = 3 mm/0.3 mm, interval = 0.3 mm, field of vision = 200 mm, matrix = 288 × 256, and NEX = 2), and pelvic DWI (diffusion sensitivity coefficient B value = 0, 800 s/mm2, TR = 2800 ms, TE = 71 ms, layer thickness = 1 mm, layer interval = 1 mm, field of vision = 340 mm, and matrix = 128 × 128). For enhanced MRI, 3D VIBE sequence axial scanning was used, 15 mL of the contrast agent was injected at 1.5 mL/s, and a T1WI fat suppression sequence was selected (TR/TE = 5.9/1.7 ms, layer thickness/layer spacing = 4 mm/0.9 mm, interval = 0.9 mm, field of vision = 320 mm, matrix = 288 × 192, NEX = 1, and dynamic scanning without interval for a total of eight phases). Siemens Magnetom Trio Tim 3.0TMR scanner: Rapid spin echo was used in the routine pelvic scanning sequence. The following sequences were used: rectal high-resolution oblique T2WI (TR/TE = 4100/93ms,layer thickness/layer spacing = 4 mm/0.4 mm, interval = 0.0 mm,field of vision=160 mm, matrix = 256 × 256,NEX = 2),and pelvic DWI (diffusion sensitivity coefficient B value = 0,600,1000,2000,3000s/mm2),TR = 3100 ms, TE = 74 ms, layer thickness = 5 mm, layer interval = 0mm, field of vision = 138×192mm, and matrix = 256×256).For enhanced MRI, 3D VIBE sequence axial scanning was used, 1.5 mL of the contrast agent was injected at 3.0mL/s, and a T1WI fat suppression sequence was selected(TR/TE = 5.68/1.72ms, layer thickness/layer spacing = 4 mm/0.9 mm, interval = 0.9 mm, field of vision = 260 mm, matrix = 288 × 192, NEX = 1, and dynamic scanning without interval for a total of thirty-five phases).

Immediately after the end of the first phase scan, 15 mL of glumine gadolinium was injected through the cubital vein mass, and continuously enhanced phase scanning was performed for the second to eighth phases 20 seconds later; each phase lasted for 20 seconds. A coronal sagittal scan was performed during the delayed period. After scanning, the image was transferred to the picture archiving and communication systems. The Functool 9.4.05 software (GE Healthcare, Chicago, IL, USA) and Siemens Sygno Tissue 4D (Siemens Leonardo, Munich, Germany) were used to process the apparent diffusion coeffificient (ADC) graph.

### 2.3 MRI morphological measurements

**Figure 1:**
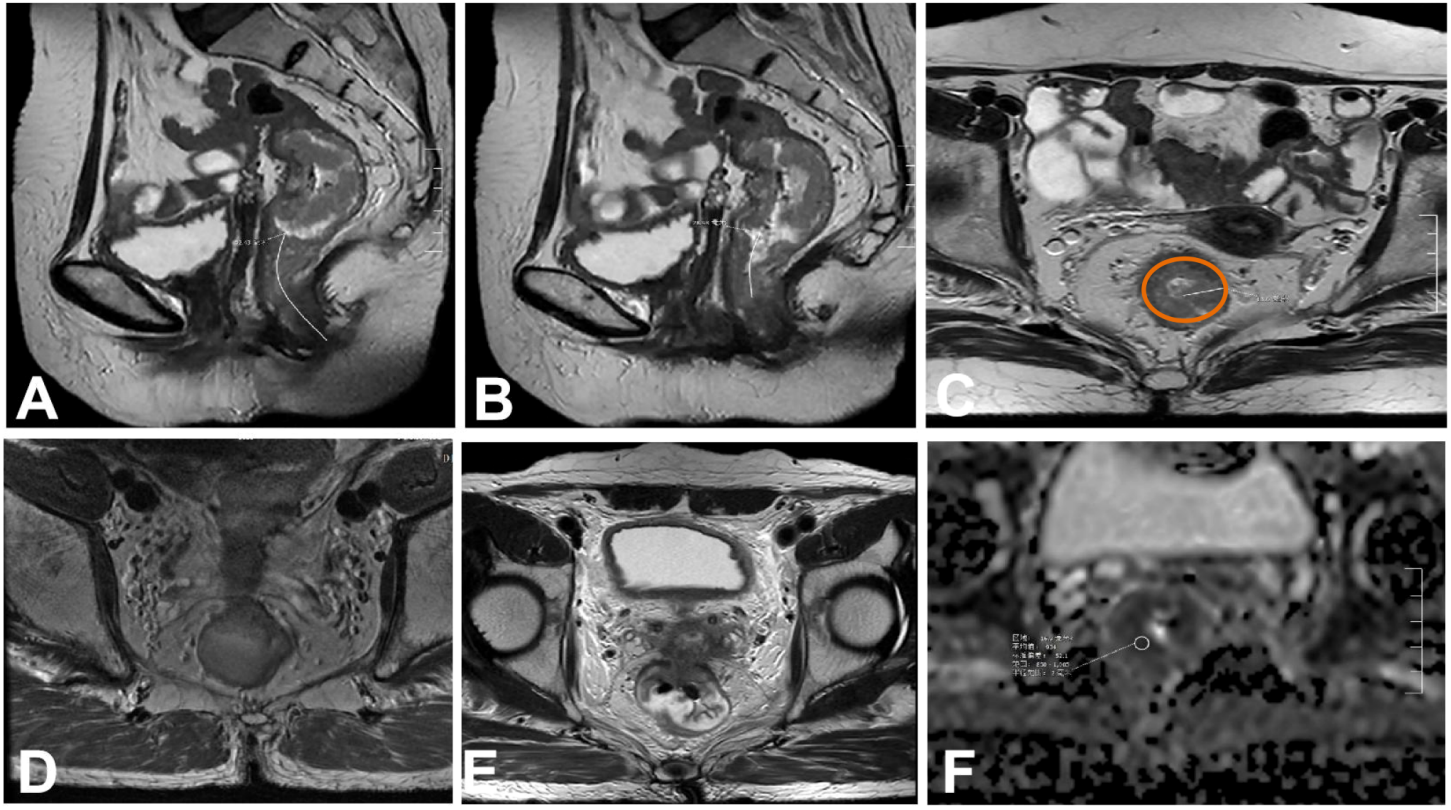
Tumor morphology detected on magnetic resonance images. **(A)** Distance from the lower margin of the tumor to the anal margin. **(B)** Distance between the lower tumor margin and the anorectal ring. **(C)** Maximum tumor diameter.The brown outline identify of the intestinal wall. **(D)** Proportion of the tumor in relation to the circumference of the intestinal wall. **(E)** Involvement of the circumferential resection margin. **(F)** apparent diffusion coeffificient (ADC) value of the lesion was measured.

Magnetic resonance images were analyzed, and the data were independently reviewed and recorded by two senior attending physicians with more than 10 years of experience in abdominal disease imaging diagnosis who were blinded to the patients’ pathological results. In cases of disagreement, the opinion after discussion was taken as the final conclusion. The final decision was made by a senior radiologist with 10 years of experience in abdominal disease imaging diagnosis and calculating intraclass correlation coefficients (ICC). The physicians assessed tumor location from the anal verge, mrT stage, tumor proportion of intestinal wall, maximum tumor diameter, circumferential resection margin, tumor location from the anorectal ring, superior rectal vein width, ADC value, T2WI EMVI score, DWI, and DCE-MRI EMVI scores. The morphological indices measured in this study were as follows and are summarized in **Figure 1**.

#### 2.3.1 Distance between the lower tumor margin and the anal margin

First, look for the lowest edge of the tumor on the sagittal section, and then measure the distance from the lower edge of the tumor to the anal margin along the central axis of the rectum. If the tumor invades the perianal region, the distance is 0. To improve readability, the “tumor position,” namely, the distance between the lower tumor margin and the anal margin is recorded (**Figure 1A**).

#### 2.3.2 Distance between the lower tumor margin and the anorectal ring

First, look for the lowest edge of the tumor on the sagittal section, and then measure the distance from the lower edge of the tumor to the anorectal ring along the central axis of the rectum. If the tumor invades the perianal region, the distance is 0 (**Figure 1B**).

#### 2.3.3 Maximum tumor diameter

The largest section of the tumor was found on the oblique axial position (perpendicular to the tumor line and scanned by MRI). The tumor was measured in a straight line perpendicular to the intestinal wall from the outer edge of the tumor. This measurement should not be performed in the conventional axial position. When the direction of the intestinal canal where the tumor is located is not perpendicular to the central axis of the human body, the scanning direction obliquely scans the focus of the tumor, resulting in a deviation in the shape of the tumor (**Figure 1C**).

#### 2.3.4 Tumor proportion of intestinal wall

The ratio of the circumferential length of the intestinal wall to the total perimeter of the intestinal wall was calculated by measuring the circumferential ratio of the tumor to the intestinal wall on the oblique axis and calculating the ratio of the circumferential length of the invaded intestinal wall to the total perimeter of the intestinal wall. This value can be divided into 0–25%, 26–50%, 51–75%, and 76–100% (**Figure 1D**).

#### 2.3.5 mrT staging

We referred to the relationship between MRI manifestations of the degree of local invasion of the rectal tumor and pathological T staging standards defined by Yingshi S et al. (21).

#### 2.3.6 Circumferential resection margin

In order to avoid observation errors, the distance between the tumor margin, metastatic lymph nodes, or tumor deposits and the mesorectal fascia is<1mm or mesorectum fascia invasion, interruption, and enhancement, the circumferential incisal margin is considered to be positive (**Figure 1E**).

#### 2.3.7 Superior rectal vein width

For upper rectal vein diameter measurement, enhanced sequence stage 3 was used as the venous stage (60 s after injection of contrast agent). At this time, the superior rectal vein was clearer, and the superior rectal artery was visible. Therefore, we used the average of three measurements of enhanced scanning at the coronal and sagittal positions of the second sacral vertebra plane (22–24).

#### 2.3.8 Apparent diffusion coefficient

We manually outlined the region of interest (ROI) on the ADC map, which had an area of approximately 20 ± 3mm^2^, and, in combination with high-resolution T2WI, DWI, and ADC images, the ROI was placed at the lowest ADC value corresponding to the highest DWI signal and the most obvious enhancement area of the lesion. Then, the ROIs of patients assessed as mrEMVI+ were plotted at the corresponding positive level, and the ROIs of patients assessed as mrEMVI- were measured at the maximum level of the tumor. We avoided areas of necrosis, blood vessels, and artifacts as much as possible. The ROI of each tumor was recorded as the average of three measurements (**Figure 1F**).

#### 2.3.9 T2WI EMVI score

For the score of the EMVI of T2WI sequences, we referred to the scoring system proposed by Smith et al. (25).

#### 2.3.10 DWI EMVI score

The EMVI scores of DWI sequences were assessed using the evaluation principles of Ahn et al. (26). A moderate or high tumor signal in normal and mildly dilated extramura vessels adjacent to the primary tumor on DWI were considered indicative of EMVI.

#### 2.3.11 DCE-MRI EMVI scores

The score of the EMVI of DCE-MRI was evaluated using the scoring system proposed by Liu et al. (27).

### 2.4 Pathological diagnosis

The rectal tumor tissues of all patients were completely removed by surgery, and the postoperative specimens were soaked in 10% formaldehyde for fixation. Representative tissues were selected, dehydrated, and embedded. Then, the embedded tissue was made into wax blocks and sliced. Based on the pathological results, wax blocks containing typical diseased tissue were resectioned and processed for Immunohistochemical examination. All sections were reviewed and evaluated by two pathologists with more than 10 years of experience each. When their assessments did not agree, they came to a consensus after discussion.

We also assessed EMVI pathologically. Pathological EMVI positivity was defined as tumor cells directly surrounding and invading the vascular or lymphatic walls or tumor cells invading the vascular or lymphatic lumen to form tumor emboli in HE-stained samples. On immunohistochemistry, the vessel walls were CD34-positive. Pathological EMVI negativity was defined as no tumor cells directly surrounding or invading the vascular or lymphatic walls in HE-stained samples and no tumor cells invading the vascular or lymphatic lumens (28).

### 2.5 Development and verification of statistical methods and prediction model

SPSS 21.0 (IBM Corp., Armonk, NY, USA) and R (version 4.0.1, http://www.rproject.org) were used for data statistics, and R, GraphPad Prism 8.3.0 (GraphPad, San Diego, CA, USA), and Medcalc 18.2.1 (https://www.mdcalc.com/) were used for image rendering. All measurement data were tested for distribution normality and homogeneity of variance. If the measurement data followed a normal or approximately normal distribution, they were expressed as mean ± SD, and a comparison between groups was performed by t-test. If there was a non-normal distribution or non-homogeneity of variance, data were expressed in M (P25, P75) form, one-way analysis of variance was used to compare means of multiple independent samples, the least significant difference method was used to compare pairs between groups, and enumeration data are expressed as rates or percentages. The chi-square test or Fisher’s exact probability method was used to compare rates of two or more independent samples. A rank-sum test was used for data where the horizontal axis represents the measure of patient or physician preference, and the vertical axis represents the net benefit rate. Decision curve analysis was performed using the R package “DCA.r.” *P* < 0.05 indicated statistical significance.

## 3 Results

### 3.1 Univariate analysis of the internal training group

**Figure 2:**
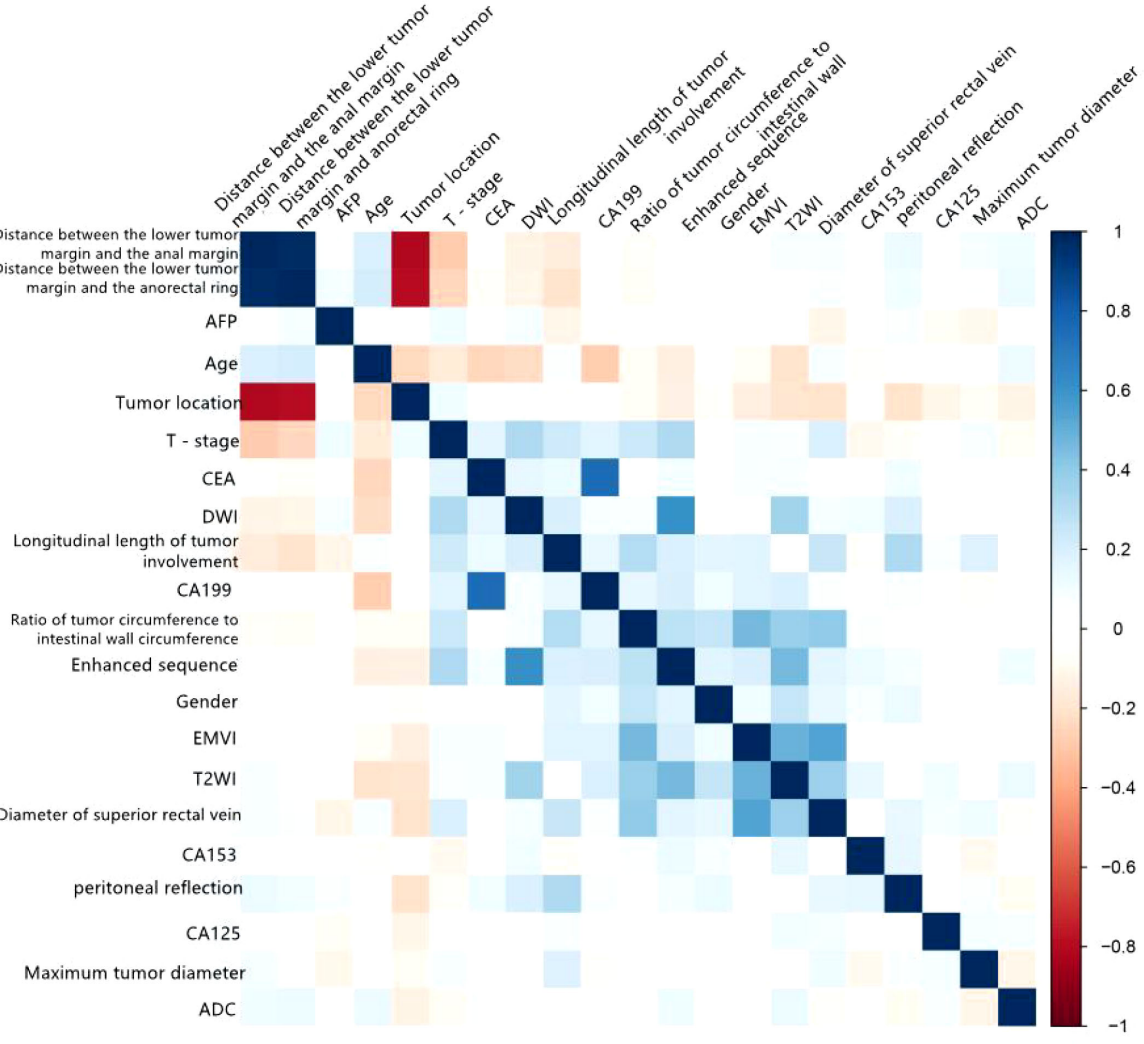
Correlation matrix of various variables with EMVI of rectal cancer for internal training group.

A total of 104 rectal cancer patients were included in the internal training group, comprising 57 men (54.8%) and 47 women (45.2%) with a median age of 61.5 years (range, 30–86 years). Pathology confirmed that 55 cases were pEMVI-positive (pEMVI+), and 49 were pEMVI-negative (pEMVI-). There were no significant differences in age, sex, height, weight, or family history of rectal cancer between the two groups *(P* > 0.05). The CEA levels of the pEMVI+ and pEMVI- groups were 14.8 ng/mL and 7.86 ng/mL, respectively, and the corresponding CA19-9 levels were 55.9 U/mL and 9.37 U/mL, respectively. There were statistically significant differences in the CEA and CA19-9 levels between the two groups. There were no significant differences in other tumor markers (AFP, CA153, and CA125) (*P* > 0.05). The differences between the tumor proportion of the intestinal wall, superior rectal vein width, and T2WI EMVI score between the pEMVI+ group and pEMVI- group was significant. There were no statistically significant differences between the two groups’ mrT stage, maximum tumor diameter, circumferential resection margin, tumor location from the anal verge, tumor location from the anorectal ring, ADC value, DWI EMVI score, and DCE-MRI EMVI score (**Table 1**; *P* > 0.05). Tumor proportion of intestinal wall, superior rectal vein width, and T2WI score was positively correlated with the EMVI of rectal cancer (**Figure 2**).The agreement between the two senior attending physicians on the selected radiological characteristics was considered good (ICC range:0.784-0.881, *P* < 0.05).

**Table 1:** General data analysis between the EMVI positive group and EMVI negative group of rectal cancer internal training group.

Characteristics	pEMVI+	pEMVI-	*P* Value
Number	N=55(%)	N=49(%)	0.46
Age (years, mean ± SD)	59.6 (10.9)	61.3 (11.7)	
Gender (%)			0.352
Male	33 (60.0%)	24 (49.0%)	
Female	22 (40.0%)	25 (51.0%)	
Family history of rectal cancer (%)			0.620
YES	3 (5.5%)	1 (2%)	
NO	52 (94.5%)	48 (98%)	
Height (cm)	161.5 (8.48)	158.27 (8.45)	0.054
Weight (kg)	58.07 (8.84)	57.04 (10.48)	0.587
CEA (ng/ml)	14.8 (47.0)	7.86 (31.4)	0.005
AFP (ng/ml)	3.18 (2.36)	3.18 (2.80)	0.991
CA125 (U/ml)	10.4 (5.71)	10.8 (6.02)	0.767
CA153 (U/ml)	11.1 (4.92)	11.5 (17.1)	0.866
CA199 (U/ml)	55.9 (183)	9.37 (10.9)	0.029
Tumor location from anal verge (cm, mean ± SD)	6.272 ± 3.5831	6.216 ± 3.2935	0.221
Tumor location from anorectal ring (cm, mean ± SD)	3.813 ± 3.6229	3.789 ± 3.5831	0.357
Tumor proportion of intestinal wall			<0.001
0~25%	2 (3.64)	4 (8.16)	
26~50%	11 (20.00)	26 (53.06)	
51~75%	23 (41,81)	12 (24.49)	
76~100%	19 (34.55)	7 (14.29)	
mrT stage(%)			0.136
T1	0	0	
T2	16 (29.1%)	22 (44.9%)	
T3	32 (58.2%)	19 (38.8%)	
T4	7 (12.7%)	8 (16.3%)	
Maximum tumor diameter (cm, mean ± SD)	1.69 (0.62)	1.74 (0.80)	0.711
Tumor length (cm, mean ± SD)	4.71 (1.85)	4.12 (1.50)	0.077
circumferential resection margin(%)			0.889
Negative	33 (60.0%)	31 (63.3%)	
Positive	22 (40.0%)	18 (36.7%)	
Tumor location from anal verge (cm, mean ± SD)	7.97 (3.33)	7.76 (3.36)	0.748
Tumor location from anorectal ring (cm, mean ± SD)	4.55 (3.27)	4.58 (3.06)	0.971
superior rectal vein width (cm, mean ± SD)	0.42 (0.06)	0.33 (0.05)	<0.001
≤0.36	12 (21.8%)	38 (77.6%)	
>0.36	43 (78.2%)	11 (22.4%)	
ADC value (×10-3mm2/s)	0.93 (0.13)	0.92 (0.20)	0.868
T2WI EMV score			<0.001
Negative (0, 1, 2)	11 (20.0%)	45 (91.8%)	
Positive (3, 4)	44 (80.0%)	4 (8.16%)	0.799
DWI EMVI score			
Negative	29 (52.7%)	28 (57.1%)	
Positive	26 (47.3%)	21 (42.9%)	
DCE-MRI EMVI score			0.053
Negative (0, 1, 2)	28 (50.9%)	35 (71.4%)	
Positive (3, 4)	27 (49.1%)	14 (28.6%)	

CEA, carcinoembryonic antigen; AFP, alpha fetoprotein; CA125, carbohydrate antigen-125; CA153, carbohydrate antigen-153; CA199, carbohydrate antigen-199; CRM, circumferential resection margin; ADC value, apparent diffusion coefficient value; T2WI EMVI score, T2-weighted imaging extramural vascular invasion score; DWI EMVI score, Diffusion-weighted imaging extramural vascular invasion score; DCE-MR EMVI score, dynamic contrast-enhanced magnetic resonance imaging extramural vascular invasion score; mrT stage, magnetic resonance imaging T stage.

pEMVI+, Positive pathological extramural vascular invasion; pEMVI-,negative pathological extramural vascular invasion.

Tumor location from the anal verge, the distance between the lower margin of the tumor and the anal margin.

Tumor location from the anorectal ring, the distance between the lower margin of the tumor and the anorectal ring.

### 3.2 ROC curve for the internal training group

**Figure 3:**
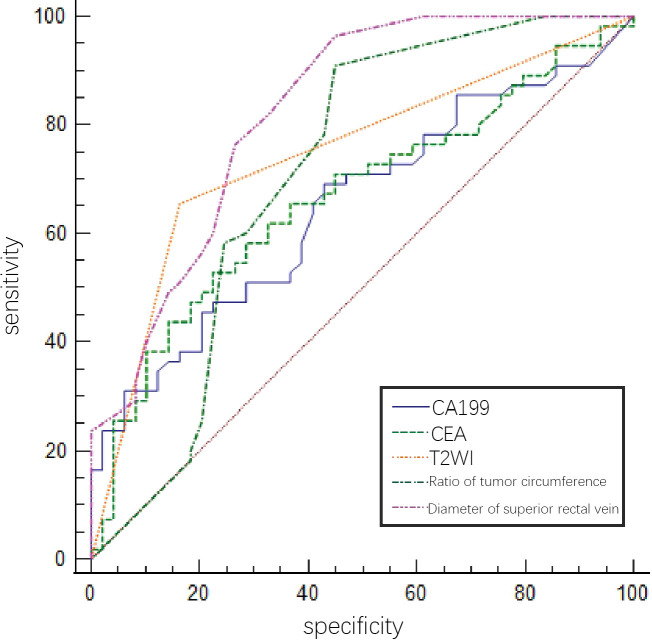
ROC of tumor markers and MRI imaging signs in predicting EMVI of rectal cancer internal training group by a single factor.

The predictive efficacies of tumor markers and imaging characteristics were selected for ROC curve analysis. The diagnostic efficiency of superior rectal vein width is relatively good. The AUC of the superior rectal vein width for predicting pEMVI was 0.824, the sensitivity was 81.82%, and the specificity was 67.35% (**Table 2** and **Figure 3**).

**Table 2:** Single factor analysis of meaningful indicators to predict the efficacy of EMVI in the rectal cancer internal training group.

Characteristics	AUC	SE (%)	SP (%)
CEA (ng/ml)	0.665	52.73	77.55
CA199 (U/ml)	0.650	69.09	57.14
Superior rectal vein width (cm, mean ± SD)	0.824	81.82	67.35
Tumor proportion of intestinal wall	0.712	90.91	55.10
T2WI EMV score	0.746	65.45	83.67

CEA, carcinoembryonic antigen; CA199, carbohydrate antigen-199; T2WI EMVI score, T2-weighted imaging extramural vascular invasion score.

### 3.3 Construction and evaluation of the nomogram for the internal training group

**Figure 4:**
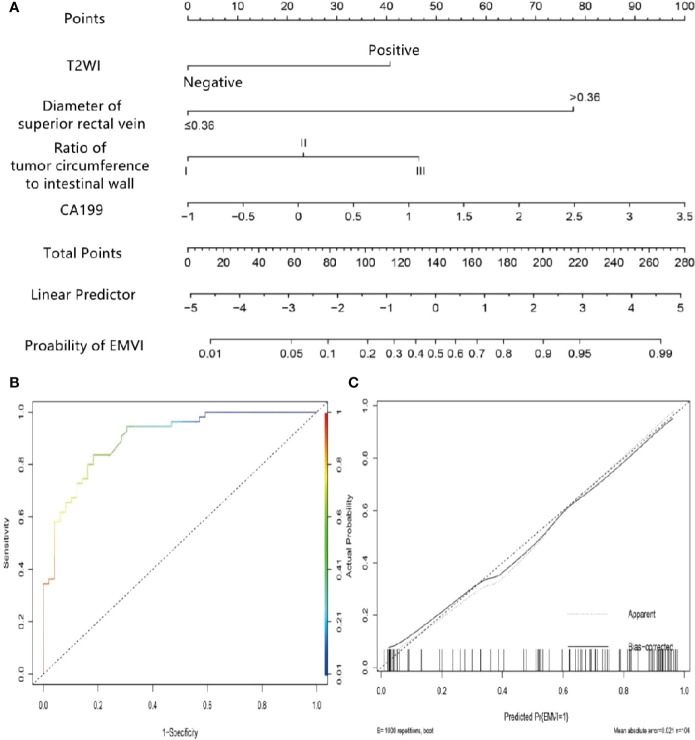
Nomogram of predicting extramural vascular invasion in the internal training group **(A)** Internal training cohort ROC curve of the nomogram **(B)** Internal training cohort calibration curve of the nomogram **(C)**.

Multivariate logistic regression analysis showed that the tumor proportion of the intestinal wall, superior rectal vein width, T2WI score, and CA19-9 were independent predictors of EMVI in the rectal cancer internal training group (**Table 3**). The variance inflation factor values for these predictors were 1.01, 1.02, 1.06, and 1.07, respectively. The error rate of the constructed logistic regression model was 19.23%, indicating that the model had good prediction efficiency, respectively. The corresponding tolerances were > 0.1 for each independent risk factor. The multiple correlations did not affect the least square estimation, and the logistic regression results were reliable. The C-index of the nomogram model was 0.899. The AUC under the ROC curve, sensitivities, and specificities of the nomogram were 0.746, 65.45%, and 83.67% for the internal training group, respectively. The calibration curve results showed that the predicted value of the model and the actual observed value fell near the 45° line, and the mean absolute error (MAE) was 0.021, indicating that the nomogram was favorably calibrated in the internal training group. In the quantitative evaluation of the calibration degree, the Brier score was 0.129, which indicated that the nomogram had good calibration ability and that there was no over-fitting (**Figure 4**) (29).

**Table 3:** Multivariate analysis between the EMVI positive group and EMVI negative group of rectal cancer internal training group.

Characteristics	β Value	S.E	Wals Z	OR Value (95%CI)	*P* Value
CEA (ng/ml)	0.9638	0.6352	1.52	2.62 (0.76-9.44)	0.1292
CA199 (U/ml)	0.7446	0.3784	1.97	2.11 (1.02-4.58)	0.0491
Tumor proportion of intestinal wall	0.9449	0.3698	2.56	2.57 (1.27-5.53)	0.0106
superior rectal vein width (cm, mean ± SD)	2.6843	0.8363	3.21	14.65 (3.36-103.80)	0.0013
T2WI EMV score	1.3822	0.5893	2.35	3.98 (1.29-13.34)	0.0190

CEA, carcinoembryonic antigen; CA199, carbohydrate antigen-199; T2WI EMVI score, T2-weighted imaging extramural vascular invasion score.

### 3.4 Verification and clinical application of the nomogram

**Figure 5:**
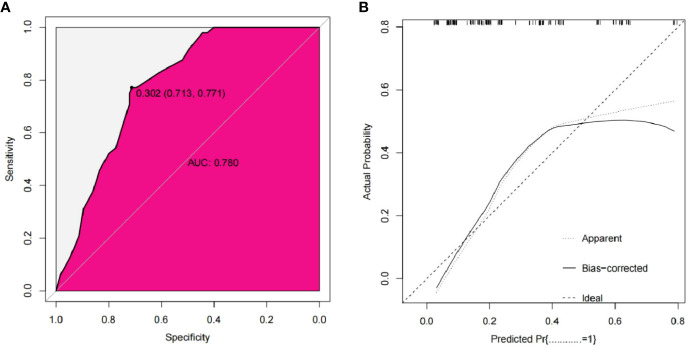
External validation cohort ROC curve of the nomogram **(A)** External validation cohort calibration curve of the nomogram **(B)**.

The bootstrap method was used for external verification, and bootstrap re-sampling was carried out 1000 times. The results between iterations remained unchanged. The AUC of the nomogram in the external validation group was 0.780, the sensitivity was 77.1%, and the specificity was 71.3%. The MAE of the calibration curve in the external validation cohort was 0.035, indicating that the nomogram was also favorably calibrated in the external validation group. The Hosmer-Lemeshow test had a *P* value of 0.150, indicating that there was no over-fitting (**Figure 5**).

The scores for each predictor were as follows: T2WI score, negative, 0 points and positive, 40 points; superior rectal vein width, ≤0.36 cm, 0 points, and >0.36 cm, 77.5 points; tumor proportion of intestinal wall 0~25%, 0 points, 26~50%, 23 points, and 50%~100%, 47.5 points; and CA19-9, 1, 15 points, 2, 67.5 points, and 3, 90 points. The preoperative risk of EMVI was calculated by adding each factor’s score to the column chart projection. For instance, if a patient had a positive T2WI score, superior rectal vein width >0.36 cm, a tumor proportion of intestinal wall, and a CA19-9 level of 3, then the total score of the nomogram was 255, and the probability of vascular invasion outside the colorectal wall would be approximately 97%.

## 4 Discussion

The purpose of this study was to explore the feasibility of using MRI characteristics to construct a nomogram to predict the EMVI in rectal cancer, which would provide valuable reference information to formulate individualized treatment plans and evaluate prognosis. Hermunen et al. evaluated that CEA and CA19-9 levels can be used as a marker for neoadjuvant treatment of advanced colorectal cancer and suggest postoperative local recurrence after combining surgical treatment (30). These MRI characteristics (e.g., tumor proportion of intestinal wall, superior rectal vein width, and T2WI score), as well as CA19-9, were independent predictive factors of EMVI in rectal cancer and were included in the nomogram. The AUC under the ROC curve, sensitivities, and specificities of the nomogram were 0.746, 65.45%, and 83.67% for the internal training group, respectively, indicating high clinical application value. Both internal and external verification indicated that the nomogram had a good differential diagnosis and calibration abilities. The total score of the nomogram was 255, and the probability of prediction was 97%, indicating an increased probability of EMVI, suggesting that more active treatment should be administered to bring more clinical benefits to patients.

Our results first show that valuable information about the blood vessels invasion outside the wall of rectal cancer is included in the tumor markers and is available from the tumor hematologic examination. CEA and CA19-9 are the most common and convenient preoperative serological indicators for colorectal cancer. These markers reflect the occurrence, development, and differentiation of tumor cells and tissues and provide important reference information for clinical diagnosis, classification, and prognosis evaluation (31). MRI characteristics of rectal cancer combined with CEA and CA19-9 can significantly improve preoperative T and N staging accuracy. However, there is no clear correlation with EMVI status (30). There were aspects of our study that differed from their work. We showed that CEA and CA19-9 levels were significantly higher in the pEMVI+ group than in the pEMVI- group (*P*=0.005, 0.029). CA19-9 was an independent prognostic factor for predicting EMVI in rectal cancer. This may be because most patients in the pEMVI- group of T stage were T3 and T4. Our results first show that valuable information about the blood vessels outside the wall of rectal cancer is included in the tumor markers and is available from the tumor hematologic examination. Tumor infiltration into the subserous membrane and adjacent mesentery and abundant peritumoral blood vessels and collateral circulation can lead to increases in serum CEA and CA19-9 (32). This study also confirmed that CEA and CA19-9 levels increased with tumor local invasion depth (T stage).

The evaluation of the vascular invasion outside the rectal cancer wall is basically to observe the relationship between the tumor and the invaded blood vessels, including the invaded blood vessel diameter, signal changes, and thrombosis. While our study mainly focused on observing the relationship between the MRI findings of the tumor itself and the superior rectal vein and the vascular invasion outside the wall of rectal cancer.

The pathological manifestations of vessel invasion in rectal cancer include invasion of vessels in the muscularis propria of the intestinal wall by cancer tissues or cells and tumor thrombi in the lumen, Vessel invasion is closely related not only to clinical stage, distant metastasis, and treatment but also to prognosis and survival (33). Papaccio et al. recommend preoperative neoadjuvant therapy, radical surgery, and additional postoperative therapy for mrEMVI-positive rectal cancer patients. This treatment regimen can effectively reduce postoperative recurrence and distant metastasis and prolong disease-free survival. Therefore, accurate preoperative assessment of EMVI status by MRI is particularly important in rectal cancer (34). In this study, pEMVI positivity was associated with the tumor proportion of intestinal wall, CEA, CA19-9, upper rectal vein diameter, T2WI sequence score, and other factors. Among them, tumor proportion of intestinal wall, upper rectal vein diameter, T2WI sequence EMVI score, and CA19-9 were independent predictive factors of EMVI in rectal cancer.

In previous studies, the evaluation of the vascular invasion outside the rectal cancer wall is basically to observe the relationship between the tumor and the invaded blood vessels, including the invaded blood vessel diameter, signal changes, and thrombosis (35–37). While our study mainly focused on observing the relationship between the MRI findings of the tumor itself and the superior rectal vein and the vascular invasion outside the wall of rectal cancer. We found that tumor proportion of intestinal wall was associated with pEMVI positivity. The MRI range increases in tumors involving the bowel, and there is an easier infringement of intestinal wall blood vessels. Further, it is easier to identify primary focal EMVI and tumor angiogenesis within the walls of the intestines. This is because blood vessels build collateral circulation, and tumor cells from the primary tumor foci use these new blood vessels to migrate through the bowel wall and metastasize (38). In addition, the diameter of the superior rectal vein was significantly larger in patients with EMVI, and the optimal cutoff value for predicting EMVI was 0.36 cm. Therefore, when a rectal tumor invades the vessels, this can be assessed by observing the superior rectal vein, which is the direct drainage vein of rectal tumors, by MRI. Rectal tumors have abundant trophoblast vessels and collateral circulation, which inevitably increase the blood flow to the reflux vein (39). Another factor may involve hemodynamics. The arteriovenous shunts and arteriovenous fistulas in the tumor also increase the blood flow of draining veins (40). This may explain why thickening and dilation of the superior rectal vein can affect the occurrence of rectal EMVI.

A shift from the slow growth of blood vessels to the rapid formation of new blood vessels indicates increased tumor growth. MRI can be used to assess the tumor proportion of intestinal wall, T2WI sequence EMVI score, and diameter of the upper rectal vein, which can indirectly reflect the local aggressiveness and blood flow to the tumor. Increased tumor neovascularization and continuous enrichment of collateral circulation increase the likelihood of cancer cell invasion into the outer muscularis propria vessels of the intestinal wall and the occurrence of cancer thrombi in the lumen (41, 42). Therefore, differences in the tumor proportion of intestinal wall, T2WI sequence EMVI score, and diameter of the superior rectal vein may lead to different EMVI outcomes (43). In addition, an increase in invasive tumor involvement in the intestinal wall leads to an increase in new blood vessel formation. However, if the basement membrane development of new blood vessels is not complete, this can increase the endothelial gap, which in turn increases permeability and allows tumor cells to traverse the adjacent intestinal wall or lumen to the muscularis propria, potentially forming tumor emboli (44). We have begun to consider whether the evaluation content of vascular invasion outside the wall of rectal cancer can meet the clinical needs and whether the content of our study can further enrich and improve the existing scoring criteria. It is hoped that the “external wall vascular invasion scoring system and model” is more reasonably perfect and that the “external wall blood vessel invasion scoring system and model” can be applied in clinical practice and it can provide more reasonable and specific guidance information for rectal cancer patients.

A nomogram is a visualization tool used to optimize statistical models for the accuracy of individual predictions. The AUC, sensitivity, and specificity of the nomogram in the validation group were 0.812, 88.9%, and 78.3%, respectively. In this study, the AUCs, sensitivities, and specificities of the nomogram constructed based on MRI features and CA19-9 were 0.899, 81.6%, and 83.6% in the internal training group, respectively, and 0.814, 87.9%, and 74.2% in the external validation group, respectively. Therefore, our nomogram had similar predictive abilities to that of Yu et al. (45). However, the factors included in this study were simpler, and we used only preoperative MRI imaging characterize and clinical-related indicators to construct the nomogram, which can save examination time and cost and be more conducive to the rational utilization and allocation of medical resources. The results of this study suggest that our nomogram can be used to assist clinical decision-making in achieving individualized and precise treatment. The individual risk of pEMVI+ rectal cancer can be estimated using the nomogram established in this study after baseline MRI examination. For low-risk patients with an overall score ≤255, less intensive treatment is needed. In contrast, high clinical attention should be paid to patients with a high risk of EMVI (total score >255), and more emphasis and intervention should be given to the formulation of individual treatment plans and prognosis evaluation.

The evaluation of the EMVI status of rectal cancer patients by preoperative pelvic MRI has become one of the important components of imaging evaluation. We believe that only by achieving multidisciplinary unity and cooperation can we find effective solutions for predicting and improving patient prognosis. New technologies and ideas may increase our understanding of the diagnosis and treatment of colorectal cancer and lead to a more accurate judgment of complex problems, such as rectal cancer imaging and pathophysiology.

This study has some limitations. First, this was a retrospective study, and there may be potential selection bias. Second, this study only extracted the imaging information from two hospitals. Although sample size considered to have more the better display effect, the information is still relatively limited. MRI studies with Multi-center and multiple parameters should be carried out in the future, such as Multi-center combined functional imaging sequences (46–48). Third, this study is a cross-sectional study, and in order to ensure the accuracy of model development and the independence of the parameters included, longitudinal studies will be the focus of our next research (49).

## 5 Conclusions

There are many influencing factors for the baseline evaluation of EMVI status in rectal cancer, and the current evaluation methods are relatively limited. Further, there is still no systematic and comprehensive evaluation method, so it is difficult to obtain evaluation results. This study constructed a nomogram model using MRI imaging characterize and clinical-related indicators that had a good predictive performance. The nomogram model is easy to use and can directly and conveniently evaluate the EMVI status of patients with rectal cancer; therefore, it has personalized preoperative predictive value for rectal cancer patients with EMVI.

## Data availability statement

The raw data supporting the conclusions of this article will be made available by the authors, without undue reservation.

## Ethics statement

The studies involving human participants were reviewed and approved by Guangxi Medical University Affiliated Cancer Hospital and The First Affiliated Hospital of Guangxi Medical University. Written informed consent for participation was not required for this study in accordance with the national legislation and the institutional requirements. Written informed consent was obtained from the individual(s) for the publication of any potentially identifiable images or data included in this article.

## Author contributions

LT: Conceptualization, Investigation, Writing-original draft, Methodology, Software, Visualization, Data curation. NL: Conceptualization, Investigation, Supervision, Writing – review & editing, Funding acquisition, Validation. SL and ZW: Supervision, Writing – review & editing, Funding acquisition, Validation. DX: Investigation, Resources. QL and CZ: Investigation, Validation. SZ, LJL, CH and LL: Investigation. All authors contributed to the article and approved the submitted version.
